# Open-label study: Exploring Culturally Adapted Psychosocial Programs for Haredi (Jewish Ultra-Orthodox) Parents of Children Exhibiting ADHD Symptoms

**DOI:** 10.1192/j.eurpsy.2025.278

**Published:** 2025-08-26

**Authors:** Y. Bloch, H. Dayan

**Affiliations:** 1 Child and adolescent outpatient clinic & Research authority, Shalvata mental health Center, Hod Hashron; 2 Department of Community Mental Health, University of Haifa, Haifa, Israel

## Abstract

**Introduction:**

Parent-focused interventions are an important part of the treatment plan for children with Attention-deficit/hyperactivity disorder (ADHD). Minority families tend to be less involved in psychosocial treatments and are more likely to discontinue the intervention. The Haredi community in Israel is a cultural-religious minority of 1.2 million people

**Objectives:**

We aimed to assess the feasibility and potential efficacy of culturally adapted psychosocial programs for attention-deficit/hyperactivity disorder (ADHD) within the Haredi community in Israel.

**Methods:**

This was an open-label non-randomized study conducted within the Haredi community in Israel between 2018 and 2021. The participants (N=265) were parents of children aged 8-12 years who had been diagnosed with ADHD or exhibited ADHD symptoms. Three culturally adapted programs were compared: 1. A program involving only the parents (N=46), 2. A program involving both children and parents (N=155) and 3. Only recorded video lectures with no interaction (N=38). Parents completed questionnaires to assess their self-efficacy using the Parenting Self-Efficacy tool (TOPSE) and their child’s ADHD symptom severity, using the Vanderbilt ADHD Diagnostic Parent Rating Scale (VADPRS) both before and after the intervention.

**Results:**

The programs demonstrated high feasibility. Of 335 referrals, 265 parents were recruited. Participation rates differed significantly between the video lectures program (60%) and the two interactive programs (80%, *p*<0.001). A significant interaction was found between time and program type for both parental efficacy (F(2,213)=5.65, p=0.004) and for ADHD symptomatology (F(2, 213)=5.65, *p*=0.004). Post hoc analysis revealed that the video lectures program showed no benefit, whereas both interactive programs (parents-only and parent-child) exhibited improvement in parental self-efficacy and reduction in reported ADHD symptom severity.

**Conclusions:**

Despite certain limitations, this study suggests that culturally adapted psychosocial programs for ADHD in the Haredi community are feasible and that including an interactive component is crucial for effectiveness.

**Disclosure of Interest:**

None Declared

